# CRISPR knockout screen implicates three genes in lysosome function

**DOI:** 10.1038/s41598-019-45939-w

**Published:** 2019-07-03

**Authors:** Guy M. Lenk, Young N. Park, Rosemary Lemons, Emma Flynn, Margaret Plank, Christen M. Frei, Michael J. Davis, Brian Gregorka, Joel A. Swanson, Miriam H. Meisler, Jacob O. Kitzman

**Affiliations:** 1 Department of Human Genetics, University of Michigan, Ann Arbor, MI 48109-5618 USA; 2 Department of Microbiology and Immunology, University of Michigan, Ann Arbor, MI 48109-5618 USA

**Keywords:** Molecular biology, High-throughput screening, Organelles

## Abstract

Defective biosynthesis of the phospholipid PI(3,5)P_2_ underlies neurological disorders characterized by cytoplasmic accumulation of large lysosome-derived vacuoles. To identify novel genetic causes of lysosomal vacuolization, we developed an assay for enlargement of the lysosome compartment that is amenable to cell sorting and pooled screens. We first demonstrated that the enlarged vacuoles that accumulate in fibroblasts lacking FIG4, a PI(3,5)P_2_ biosynthetic factor, have a hyperacidic pH compared to normal cells'. We then carried out a genome-wide knockout screen in human HAP1 cells for accumulation of acidic vesicles by FACS sorting. A pilot screen captured fifteen genes, including *VAC14,* a previously identified cause of endolysosomal vacuolization. Three genes not previously associated with lysosome dysfunction were selected to validate the screen: *C10orf35*, *LRRC8A*, and *MARCH7*. We analyzed two clonal knockout cell lines for each gene. All of the knockout lines contained enlarged acidic vesicles that were positive for LAMP2, confirming their endolysosomal origin. This assay will be useful in the future for functional evaluation of patient variants in these genes, and for a more extensive genome-wide screen for genes required for endolysosome function. This approach may also be adapted for drug screens to identify small molecules that rescue endolysosomal vacuolization.

## Introduction

In addition to its role in turnover of macromolecules, the lysosome functions in calcium storage, monitoring of metabolism, and regulation of transcription^1^. The phosphoinositide PI(3,5)P_2_ is a signaling lipid with regulatory roles in lysosome homeostasis, mediated in part by activation of ion channels in the lysosomal membrane^2–6^. Mutations of the PI(3,5)P_2_ biosynthesis genes *FIG4*, *VAC14* and *PIKFYVE* disrupt lysosome turnover, resulting in accumulation of large acidic cytoplasmic acidic vesicles bounded by membranes containing the endolysosomal membrane markers LAMP1 and LAMP2 (ref.^7^). Mutations of these genes result in the human disorders Charcot-Marie-Tooth type 4J (OMIM 611228), polymicrogyria with epilepsy (OMIM 612619), Yunis-Varón syndrome (OMIM 216340), and childhood-onset striatonigral degeneration (OMIM 617054) (refs^8–11^). Fibroblasts from patients with these disorders accumulate characteristic enlarged acidic vacuoles that are also seen *in vivo* in mouse mutants.

Forward genetic screens in yeast have identified many genes with important roles in lysosome function^12–15^. Mammalian homologs have been identified for some but not all of these, suggesting a role for as-yet unknown factors in regulating the higher eukaryotic lysosome. To identify such factors in mammalian cells, we turned to pooled CRISPR/Cas9-mediated screens which utilize gene knockout, silencing, or over-expression^16^. Despite their promise, a key bottleneck in carrying out these screens is their requirement for cellular assays specific to a phenotype, pathway, or biochemical activity of interest.

Here we describe a fluorescence activated cell sorting (FACS) assay for cells with elevated content of acidic vesicles, developed using a clonal FIG4-null human cell line which accumulates acidic vesicles. We demonstrate that this assay can support genome-wide CRISPR screening, and as a proof of principle, we newly implicate three genes in lysosome function. This screen has the potential to identify additional genes involved in lysosomal physiology that may contribute to human disease.

## Results

### Acidic pH of vacuoles in *Fig4* null mouse fibroblasts

**Figure 1:**
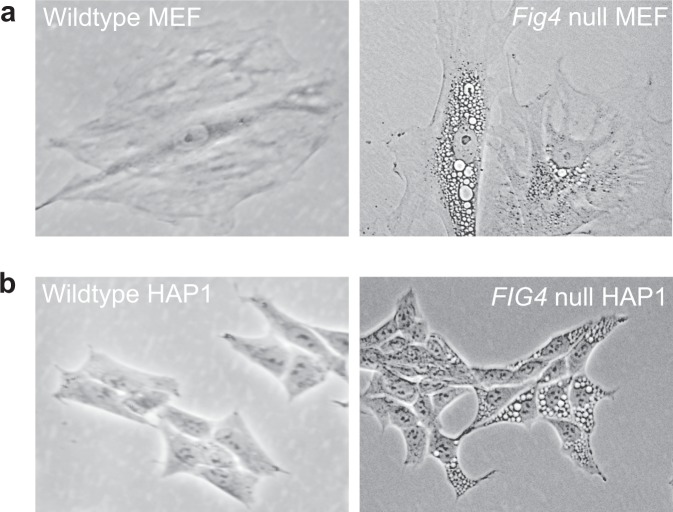
Vacuolization due to *FIG4* mutations in two cell types. (**a)** Mouse embryonic fibroblasts derived from *Fig4* null mice^8^. The number of vacuoles per cell is variable. **(b)** *Fig4* null HAP1 cell line 3D4 derived here. Wildtype cells are shown at left for comparison.

**Figure 2:**
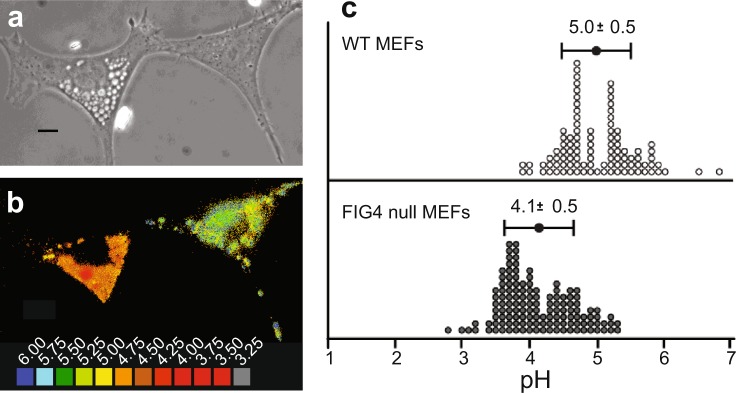
Acidic pH of vacuoles in *Fig4* null fibroblasts demonstrated by Oregon Green Dextrain staining and ratiometric fluorescence microscopy. (**a**) Phase contrast of *Fig4* null fibroblasts with vacuolated cell on the left. (**b**) Fluorescence emission at 535 nm was recorded for excitation wavelengths of 440 nm (pH insensitive, I_(ex440)_) and 488 nm (pH sensitive, I_(ex488)_). Ratiometric images were obtained as I_(ex488)_/I_(ex440)_, calibrated using separate, pH-clamped cells, and the calculated pH values were displayed as pseudocolored images. (**c**) Vesicles in *Fig4* null fibroblasts are acidic, with an average pH 4.1 ± 0.5 (n = 132) compared with 5.0 ± 0.5 (n = 99) in wildtype fibroblasts (*P* ≤ 10^−25^, t-test).

Cultured embryonic fibroblasts from *Fig4* null mice accumulate large cytoplasmic vacuoles (Fig. 1a) that contain LAMP2 and other lysosome markers^8,17,18^. We measured the pH of these vacuoles using ratiometric fluorescence^19^ by imaging with the pH-sensitive dye Oregon Green Dextran (Fig. 2a,b). Quantification of average lysosome pH was calculated as described in Methods for each cell (Fig. 2c). Lysosomes in *Fig4* null fibroblasts have an average pH of 4.1 ± 0.5 (mean ± sd), significantly more acidic than the normal lysosomes in wild-type cells (p < 10^−25^, *t*-test), whose observed pH of 5.0 ± 0.5 is comparable to previous measurements^20^. The acidity of the vesicles accumulating in *FIG4* null cells is consistent with their origin from the endolysosomal compartment, and indicates that PI(3,5)P_2_ deficiency, a known consequence of *FIG4* loss, may lead to hyperacidification of mammalian lysosomes.

### Generation of *FIG4* null HAP1 cells with an expanded acidic compartment

To generate a PI(3,5)P_2_-deficient human cell line, we targeted *FIG4* exon 6 using CRISPR/Cas9 in the haploid human cell line HAP1. We isolated two clonal *FIG4* knockout cell lines, 1C2 and 3D4, transfected with independent sgRNAs. By visual inspection, and similar to *FIG4* null mouse fibroblasts, the two clones contained a high proportion of vacuolated cells (Fig. 1b). Both clones contain frame-shifting indels in *FIG4* predicted to result in premature stop codons (Fig. S1a). Vacuolization was rescued by transfection with wildtype *FIG4* cDNA (Fig. S1b). These *FIG4* null cells and the wild-type HAP1 line from which they were derived were used to develop and optimize the flow-sorting assay for vacuolated cells.

### FACS assay to detect cells with an expanded acidic compartment

**Figure 3:**
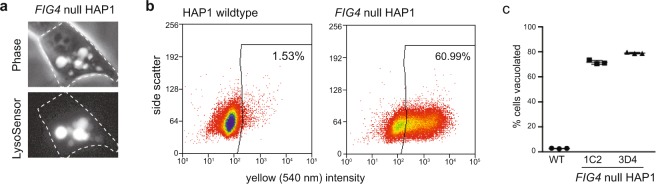
FACS assay for acidic vesicle content of cultured HAP1 cells. (**a**) *FIG4* null cells grown for 18 hours prior to staining with 5 uM LysoSensor contain large vesicles that retain the fluorescent dye. (**b)** Using stringent gating criteria based on elevated intensity of fluorescence, 61% of *FIG4* null cells were separated into a window that contains only 1.5% of wildtype cells. **(c)** Reproducible quantification of vacuolated cells from the two *FIG4* null clones 1C2 and 3D4.

**Figure 4:**
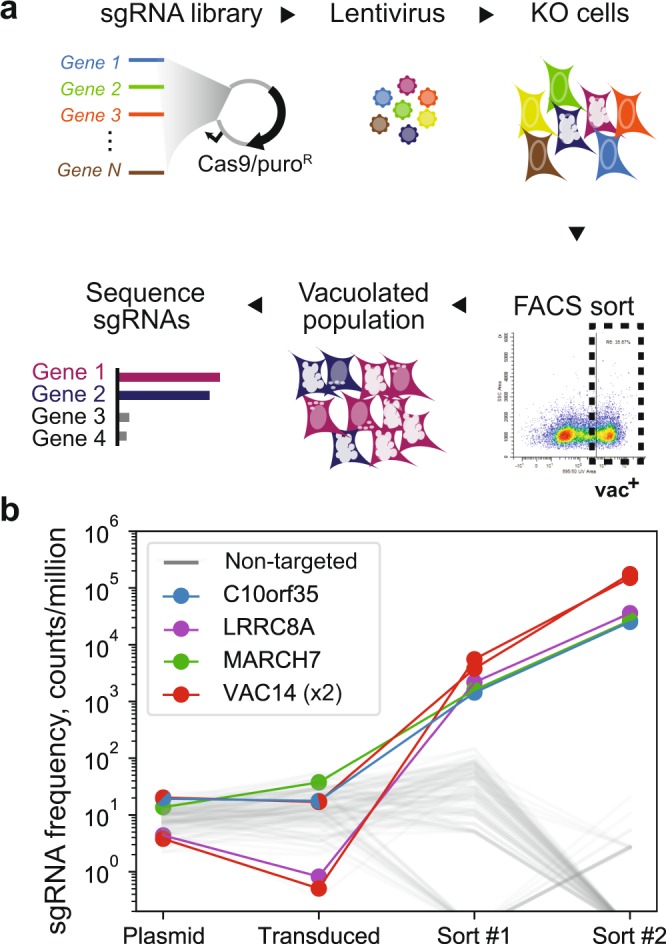
Genome-wide knockout screen. (**a**) GeCKOv2 CRISPR library^22^ was used to infect wild-type HAP1 cells at a MOI of 0.2, resulting in knock-out of a different gene in each cell. Gene knockouts resulting in vacuolated cells (vac+) were enriched by two rounds of FACS sorting. Deep sequencing identified sgRNAs and target genes knocked out in the vac+ population**. (b)** Representative data demonstrating enrichment of four genes in sorted cells. Frequency of sgRNAs (y-axis; reads per million reads) in the plasmid library, starting transduced population, after the first sort, and after the second sort. Gray lines represent 100 randomly selected non-targeted sgRNAs. The selected sgRNAs are enriched by 3 to 4 orders of magnitude (Table 1).

The fluorescent dye LysoSensor permeates the cell membrane and accumulates in lysosomes in a pH-dependent fashion^21^. After staining, vacuolated cells have elevated fluorescence when excited at 329 nm that is readily visualized in individual cells by epifluorescence microscopy (Fig. 3a). The difference between wildtype and mutant cells can be quantified within a population by fluorescence activated cell sorting (FACS). We applied stringent gating criteria that selected >60% of *FIG4* null cells as LysoSensor positive, but <2% of wildtype cells (Fig. 3b). The reproducibility of sorting results obtained from 3 experiments each on the two independent *FIG4* null clones is shown in Fig. 3c.

### A genome-wide screen for mutations causing accumulation of acidic vacuoles

We hypothesized that the FACS assay could be used as part of a mutagenic screen in order to discover novel genes which phenocopy *FIG4*-null vacuolization due to their roles in lysosome dynamics such as PI(3,5)P_2_ production. We adopted a CRISPR/Cas9-mediated pooled screening approach in which each cell in a mixed population is a knockout for a different gene (reviewed in ref.^16^). This screen involved four steps (Fig. 4): first, HAP1 cells were transduced at low multiplicity of infection (MOI) with the GECKOv2 lentiviral library^22^ encoding Cas9 nuclease and six sgRNAs per gene targeting 19,050 human genes; then, pooled cells carrying individual gene-disrupting mutations were stained with LysoSensor and vacuolated cells were captured by FACS; next, genomic DNA was isolated from pooled captured cells and integrated sgRNA sequences were amplified by PCR; finally, enriched sgRNAs were identified by sequencing of the PCR products and demonstration of elevated abundance in FACS-selected cells compared with the starting population.

### Preliminary screen identifies 15 genes enriched in vacuolated cells

Wildtype HAP1 cells were transduced with the pooled GeCKO library at an MOI of 0.2 and selected with puromycin. The mixed population was expanded as a pool to produce adequate material for downstream analysis. Sequencing sgRNAs from the plasmid pool and transduced cell population indicated that most guides were present at each step, and as expected, genes in cell-essential pathways were significantly depleted after transduction and outgrowth (Fig. S2). After staining with Lysosensor, the transduced population was sorted, using a stringent gating which captured <0.001% of cells from a wildtype cell population. From the genome-wide knockout cell population, 1.7 × 10^3^ cells were captured out of ~10^7^ sorted (0.017%), a substantial excess over the wild-type controls. These vacuolated KO cells were expanded as a pool and further sorted for one additional round to increase the purity of the vacuolated population. The resulting population contained predominantly vacuolated cells when assessed visually. Sequencing identified 16 sgRNA sequences targeting 15 genes which together accounted for 95% of the sequences (Table 1). Among these, the two most-highly enriched sgRNAs targeted *VAC14*, a gene required for PI(3,5)P_2_ biosynthesis that is known to cause vacuolization and lysosome dysfunction^11,15,23,24^. One sgRNA targeted *CLN8*, a transmembrane protein responsible for a neurodegenerative lysosomal storage disorder^25,26^. Given the identification of bona fide lysosomal factors, we focused on the remaining 13 highly enriched genes not previously associated with lysosome function (Fig. 4b).

**Table 1:** Top-ranked sgRNAs (n = 16) cumulatively accounting for 95% of all reads after two rounds’ FACS sorting for vacuolated cells.

Gene	sgRNA	Plasmid	Transduced	Sort #1	Sort #2
*VAC14*	**HGLibB_53419**	0.00948%	0.00510%	0.37784%	17.50467%
*VAC14*	**HGLibA_53485**	0.00177	0.00015	0.55616	15.01131
*XYLT1*	**HGLibB_54553**	0.00594	0.00264	0.19273	9.74554
*PYCR1*	**HGLibB_39621**	0.01257	0.00565	0.18112	9.35672
*PPBP*	**HGLibA_37855**	0.00745	0.00586	0.24632	6.64521
*ZUFSP*	**HGLibA_57060**	0.00990	0.00601	0.19631	5.78972
*SHISA9*	**HGLibB_44001**	0.00459	0.00353	0.13282	4.61743
*PGAM4*	**HGLibA_36202**	0.00415	0.00366	0.19185	3.69911
*LRRC8A*	**HGLibA_27429**	0.00204	0.00025	0.21739	3.59412
*CLN8*	**HGLibA_10085**	0.00565	0.00630	0.18717	3.52703
*P2RX2*	**HGLibA_34848**	0.01772	0.02370	0.18083	3.38788
*CMTM8*	**HGLibA_10231**	0.00921	0.00899	0.13789	2.89416
*MARCH7*	**HGLibA_28346**	0.00639	0.01131	0.15926	2.67105
*C10orf35*	**HGLibA_05070**	0.00907	0.00533	0.14351	2.52899
*MLL3*	**HGLibB_29359**	0.00614	0.00298	0.08345	1.59024
*KMT2C*	**HGLibA_25328**	0.00596	0.00279	0.08729	1.57383

Percent frequency of each guide (row) is shown at each selection timepoint (column) as a percentage of total reads.

### Functional validation of three selected genes

**Figure 5:**
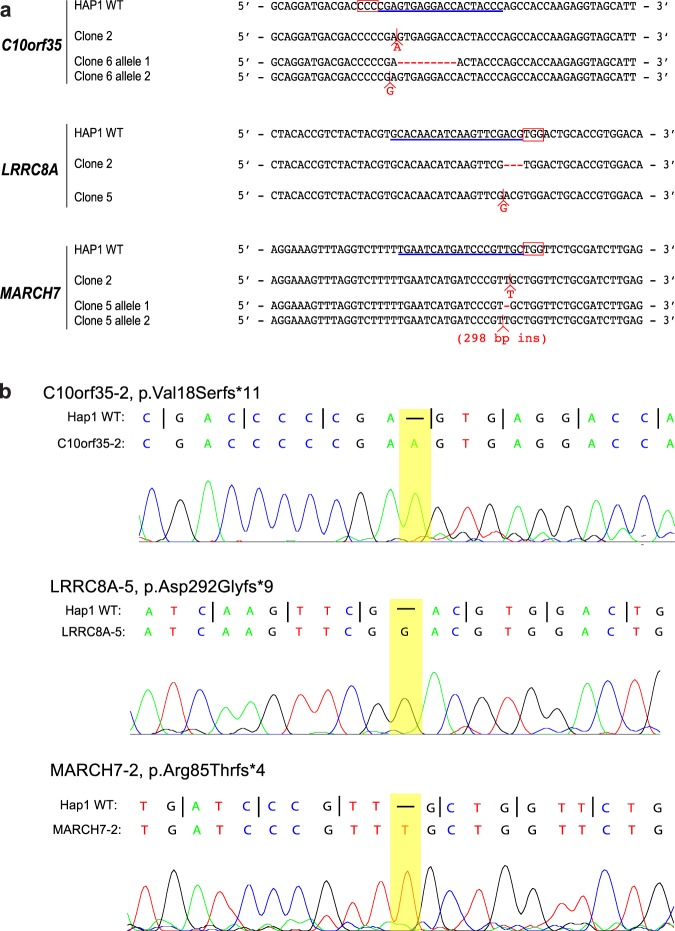
Identification of null mutations in independently targeted cell lines. (**a**) Sequence alignments around the CRISPR/Cas9 cut sites at each targeted exon in mutant clones. The sgRNA sequence is underlined in blue; the protospacer-adjacent motif (PAM) is boxed in red. Clones C10orf35-6 and MARCH7-5 are diploid and compound heterozygous for two null mutations. **(b)** DNA sequence chromatograms for the first mutant clone for each targeted gene. (Chromatograms for the second clone for each gene are provided in Fig. S3). Insertions in the mutant sequences are highlighted and represented by dashes in the wildtype sequence.

**Figure 6:**
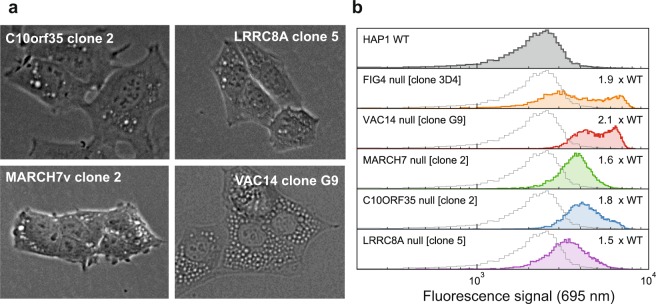
Clonal knock-out cell lines are vacuolated. (**a**) Phase contrast images reveal vacuoles in each null cell line. Scale bar: 25 µm. **(b)** Distribution of fluorescence for the parental HAP1 wildtype and five mutant cell clones. All of the mutant lines exhibit significantly elevated fluorescence due to accumulation of acidic vesicles (mean fold-change over wild-type listed; all are P < 10^−100^, two-sided Kolmogorov-Smirnov test).

**Figure 7:**
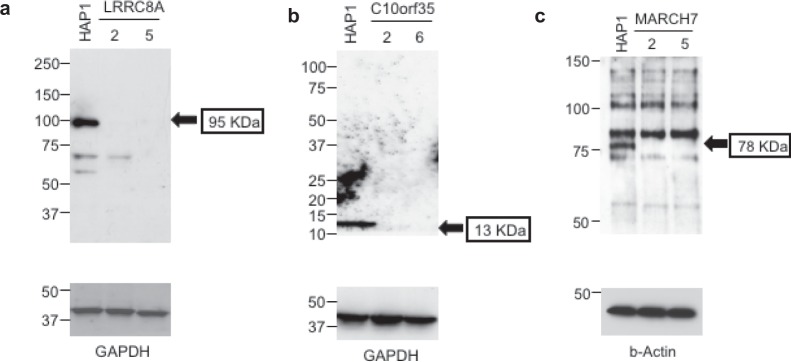
Protein analysis of HAP1 cell lines with null mutations of *C10orf35*, *LRRC8A*, and *MARCH7*. Whole cell lysates were subjected to Western blotting followed by immunostaining for **(a)** *C10orf35*, **(b)** *LRRC8A*, and **(c**) *MARCH7*. Proteins with the predicted molecular weight (arrows) were present in wildtype HAP1 cells but not in the mutant clones. MW markers are shown at the left of each gel. The internal controls GAPDH and beta-actin demonstrate comparable protein loading of wildtype and mutant lysates.

**Figure 8:**
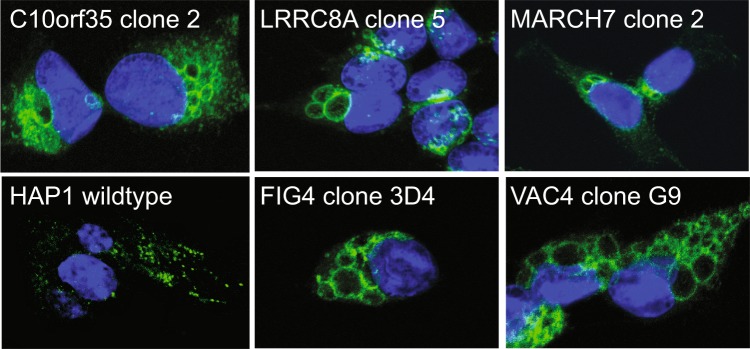
The enlarged vacuoles in mutant cells are LAMP2-positive. Confocal images of fixed cells show LAMP2 (green) localized to small puncta in wildtype cells and localized to the periphery of enlarged vesicles in mutant HAP1 cell lines, supporting the endolysosomal origin of the vesicles. Nuclei were counterstained with DAPI (blue). Scale bar: 10 µm.

*C10orf35*, *LRRC8A* and *MARCH7* were selected for validation by re-creating individual knock-out clones using CRISPR/Cas9 and two new sgRNA sequences (Table S1). *VAC14* was included as a positive control. After isolation and clonal expansion of individual transfected cells, sequencing revealed frame-shifting indel mutations predicted to result in premature truncation of each targeted gene (Figs 5 and S3). All derived mutant cell lines contained vacuolated cells visible by phase contrast microscopy (Fig. 6a), and all showed significantly greater LysoSensor staining by FACS analysis compared with wild-type cells (Fig. 6b). Both microscopy and FACS analysis indicated a lesser degree of vacuolation for *C10orf35*, *LRRC8A* and *MARCH7* knockouts compared with *FIG4* or *VAC14*, suggesting non-identical roles for these factors, or differential sensitivity to their loss. We observed complete absence of targeted protein expression in each mutant cell line by Western blotting (Fig. 7), indicating that the observed vacuolation phenotypes in these mutants result from loss of function. Finally, to confirm the endolysosomal origin of the vesicles in the mutant cell lines, we carried out immunostaining of the membrane protein LAMP2. The membrane surfaces of enlarged vacuoles in the *C10orf35*, *LRRC8A* and *MARCH7* null clones are stained for LAMP2, while wildtype cells exhibit punctate staining of small lysosomes (Fig. 8). We conclude that mutations of C10orf35, LRRC8A and MARCH7 generate enlarged vesicles derived from the endolysosomal pathway, the same origin as those observed in *FIG4* or *VAC14*-null cells.

## Discussion

Application of CRISPR-Cas9 mutagenesis to pooled screens provides a new tool for the study of gene function in cellular and animal models^16,27^. Although the capacity to programmably target entire coding or regulatory genomes represents a major advance over spontaneous or random mutagenesis, these pooled approaches share with classical forward genetic screens the requirement for an assay to identify and enrich cells with the phenotype of interest. As a result, while they can be readily applied as fitness-based selections (e.g., for increased cellular growth, or drug resistance), adapting them to other biological processes requires the development of cellular assays specific to the pathway or activity of interest. For instance, two recent screens have identified knockouts which perturb expression of an engineered reporter of Hedgehog signaling^28^, or which dysregulate native fetal hemoglobin expression^29^, necessitating specialized, sortable expression assays for each target. Here, we have developed a FACS-based assay which for the first time enables pooled screening for genes which disrupt lysosomal dynamics and result in an enlarged lysosomal compartment.

The first candidate gene arising from this screen, *LRRC8A*, encodes a component of the volume-regulated anion channel (VRAC) that regulates cell volume in response to changes in extracellular osmolarity^30–32^. Elucidation of the protein structure of LRRC8A revealed a transmembrane pore domain and a leucine-rich cytoplasmic domain^33–35^. Gene inactivation in a conditional knock-out mouse has demonstrated roles for *LRRC8A* in spermatogenesis and insulin secretion^36,37^. Our data indicate that *LRRC8A* is also involved in regulation of lysosome volume, and that loss of function leads to osmotic swelling of the lysosome. We previously proposed osmotic swelling as the basis for vacuolization in *FIG4* and *VAC14* null cells, resulting in those cases from impaired activation of lysosomal cation channels by PI(3,5)P2 (ref.^7^). It will be of interest to compare the effects of neuronal knockout of *LRRC8A* with the neurodegeneration that results from neuronal inactivation of *FIG4* (ref.^38^).

The second gene, *MARCH7*, is a member of the ubiquitin ligase E3 class that targets proteins for trafficking to the multivesicular body for degradation in the proteosome. A mutation in another ubiquitin E3 ligase is responsible for the mouse mutant mahoganoid, which also exhibits accumulation of cytoplasmic vesicles and spongiform degeneration of the CNS similar to the *FIG4* and *Vac14* null mice^39^. The under-representation of null alleles of *MARCH7* in the ExAC exome database^40^ (pLI score of 0.98) indicates that haploinsufficiency of *MARCH7* is deleterious and could result in dominantly inherited disease. Our screen also identified *C10orf35*, which encodes a predicted single-pass membrane protein whose cell localization and function have not previously been characterized.

Since it is based on LysoSensor fluorescence in an acidic environment, the FACS assay specifically detects enlarged lysosomes. The vesicles in *FIG4* null cells were hyperacidic, with a pH of 4.1 compared with 5.0 in wildtype fibroblast cells. One mechanism that could account for hyperacidity would be dependence on PI(3,5)P_2_ of a proton-permeable anion channel involved in proton countertransport. For example, impaired activation of ClC-7, proposed to play a role in escape of protons from the lysosome interior^41^, could lead to lower intralysosomal pH.

Although we functionally validated all three novel genes selected for follow-up, each was only captured once in this pilot study, suggesting that a number of additional genes with roles in lysosome dynamics remain to be discovered. We plan to expand the screen described here to achieve genome-wide saturation, to generate a catalog of genes with roles in lysosome regulation, and to further contribute to the understanding of lysosome biology in human cells. One limitation of this study is that it does not address potential tissue specificity of cytoplasmic vacuolization. While lysosomes are universal components of the mammalian cell, *in vivo* vacuolization in *FIG4* and *Vac14* mutant mice is restricted to neural tissue^17^, reflecting an apparent greater susceptibility. However, other cells derived the mutant mice exhibit vacuoles when cultured *in vitro*, including fibroblasts, osteoblasts, neurons, oligodendrocytes, and bone marrow macrophages. Additionally, as with other knock-out screens, genes which are essential for growth in this particular cell line (HAP1) would not be identified.

The FACS assay for vacuolization described here can be applied in the future to evaluation of novel patient mutations in these genes, addressing the challenge of interpreting ‘variants of unknown significance’ identified by exome and whole genome sequencing^42–44^. As a result of this preliminary screen, disruptive variants in *C10orf35, LRRC8A* and *MARCH7* found by clinical sequencing should be considered candidate genes for neuromuscular and lysosomal disorders of unknown origin. This functional assay could also be adapted to drug screening for therapeutic small molecules that reverse the vacuolization phenotype.

## Methods

### Generation of *FIG4* null HAP1 cells

Human HAP1 cells (Horizon Discovery, #C631) were originally derived from human hematopoetic cells^45,46^. HAP1 and HAP1-derived cell lines and pools were maintained in Iscove’s Modified Dulbecco’s Medium (IMDM) supplemented with 10% FBS and 100 units/mL penicillin, 100 µg/mL streptomycin (Invitrogen). Guide sequences targeting *FIG4* exon 6 were selected using E-CRISP^47^ and screened for potential off-target sites with Cas-OFFinder^48^. Guides were ordered as oligonucleotides from IDT, annealed, and cloned into plasmid pSpCas9(BB)-2A-GFP^49^ to express the sgRNA along with SpCas9 and GFP genes. HAP1 cells (~1 million) were transfected with 12 ug sgRNA plasmid using 6 ul Fugene reagent (Promega). After 18 hrs of growth, individual GFP-positive cells were flow sorted into a 96 well plate. Resulting colonies were examined under a light microscope and those with vacuolated appearance were expanded. Induced mutations in the targeted region (*FIG4* exon 6) were identified by Sanger sequencing. Clones 1C2 and 3D4 were generated with two different sgRNAs and used in subsequent experiments.

### Measurement of lysosomal pH

Ratiometric fluorescence of 10kD Oregon Green Dextran (OGDx) was carried out as previously described^19^. OGDx was included in the cell culture media for 2 hours followed by a 24 hour chase to permit accumulation of the fluorophore in lysosomes. Fluorescence emission at 535 nm was recorded for the excitation wavelengths 440 nm (pH insensitive) and 488 nm (pH sensitive). The ratio of fluorescence emission at these wavelengths was compared to a standard curve generated by incubating OGDx-labeled cells in buffers of known pH in the presence of the ionophores valinomycin and nigericin. Small punctate lysosomes and a subset of enlarged vacuoles were labeled by OGDx in *FIG4*-null cells. For each cell, a single average pH value was obtained for all of the OGDx-containing lysosomes.

### FACS assay using accumulation of fluorescent dye to separate vacuolated and wildtype cells

Eighteen hours prior to FACS analysis, HAP1 cells were plated in 100 mm plates at 40,000 cells per cm^2^ in IMDM containing 10% FBS with a final concentration of 100 units/mL penicillin, 100 µg/mL streptomycin, and 0.25 µg/mL of Gibco Amphotericin B (“full medium”). Cells were incubated in a humidified incubator at 37 °C with 5% CO_2_. The endolysosomal compartment was labeled by incubation for 15 min with 5 μM LysoSensor Yellow/Blue DND-160 dye (LysoSensor) (Molecular Probes). LysoSensor fluorescence is excited at 329 nm with emission peaks at 440 and 540 nm. Labeling medium was removed and cells were washed 3 times at room temperature with PBS. Cell monolayers were removed from the plates by treatment at 37 °C for 5 minutes with TrypLE Express Enzyme Without Phenol Red (Thermo Fisher Cat #12604013). Cells were suspended in PBS containing 2% FBS and placed on ice. Propidium iodide, final concentration 1.5 ug/ml, was added to the cell suspension as a viability marker.

### GeCKO library and lentiviral transduction

GeCKO v2 pooled knock-out libraries “A” and “B” were obtained from Addgene (#1000000048; gift of Feng Zheng). Each half-library was transformed in bulk into Endura strain *E. coli* (Lucigen), expanded in 150 ml culture, and isolated by maxiprep (Zymo Research #D2403). Lentiviral particles were produced by the University of Michigan Vector Core by co-transfecting HEK-293T cells with packaging plasmids psPAX2 and pMD2.G. For bulk transduction, wild-type HAP1 cultures were expanded to 12.5 × 10^6^ cells in T175 flasks. Viral supernatant (0.25 ml, ~10^7^ IFU/ml) was added to reach an approximate multiplicity of infection of 0.2. After 24 hours, puromycin (2.5 ug/ml final concentration) was added to the media to select for transduced cells.

### Cell sorting and sequencing

Pooled transduced HAP1 cultures were expanded to ~10^8^ cells (6–7 doublings). Cells were plated at 40,000 cells/cm^2^, stained with LysoSensor as above, and approximately 10^7^ cells were sorted. Captured cells were plated, expanded (~15 doublings), and subjected to an additional round of sorting by the same procedure to further enrich for vacuolated cells. After outgrowth (~4 doublings), genomic DNA was prepared using the Gentra Puregene kit (Qiagen #158767). Integrated sgRNAs were amplified by PCR for 24 cycles as previously described^50^, with modified amplification primers which included a randomized ‘stutter’ sequence (Table 1) to mitigate artifacts on the Illumina platform from low-diversity sequences. For sgRNA sequencing, 2 ug of gDNA per PCR reaction was used as template, and four reactions were pooled per sample to ensure sufficient representation of integrated sgRNAs. The resulting amplicon was diluted and further amplified in a second-round PCR to add Illumina adaptor sequences and sample-specific index barcodes. All libraries were pooled and sequenced on MiSeq and HiSeq instruments using 50-bp single-end reads.

### Single gene validation knock-out

sgRNAs were targeted to constitutively included exons of *C10orf35*, *LRRC8A* and *MARCH7* using Ensembl gene models. Potential sgRNA sequences were designed and cloned into pSpCas9(BB)-2A-Puro, transfected into wild-type HAP1 cells, and clonal KO cells were isolated as described above for *FIG4*.

### Western blotting

Protein was isolated from cells grown in a monolayer to 70~80% confluence using RIPA buffer and protein inhibitor cocktail (Thermo Scientific). Samples were prepared for electrophoresis under reducing conditions with Laemmli sample buffer (Bio Rad) containing 2-mercaptoethanol (Sigma Aldrich). Twenty ug of protein per lane was loaded onto acrylamide gels of varying concentration, depending on the molecular weight of the protein. The primary antibodies were mouse anti-C10orf35 (Abcam, 1/5,000 dilution), rabbit anti-LRRC8A (Thermo Scientific, 1/2000 dilution), and rabbit anti-MARCH7 (Sigma Aldrich, 1/1500 dilution). Membranes were subsequently incubated with peroxidase-labeled secondary antibodies. Chemiluminescence was detected with SuperSignal West Femto chemiluminescence reagent (Pierce, Thermo Scientific) and HyBlot-CL autoradiography film (Denville Scientific).

### Microscopy of cultured cells

To visualize vacuoles, cells were grown in full medium for 36 hours after replating, and phase contrast images were taken with the EVOS FLc system (Life Technologies). For LAMP2 immunostaining, cells were grown for 36 hours on 4-compartment slides (LAB-TEK, Rochester, NY), washed 3x with cold PBS, and fixed with cold methanol for 5 min at −20 °C. Cells were permeabilized by incubation for 10 min in 0.1% Triton X-100 in PBS and then blocked for 1 hour using 5% goat serum in PBS. Slides were processed for indirect immunofluorescence by incubation with LAMP2 antibody (Developmental Studies Hybridoma Bank, University of Iowa), 1/4000 dilution, for 2 hours at room temperature, then washed with PBS three times for 5 min each and incubated with Alexa-488 fluorescent-labeled secondary antibody (Invitrogen, 1/3000 dilution) in blocking buffer. Slides were washed again with PBS (3 times, 5 min each), and coverslips were mounted with Prolong Gold antifade reagent containing DAPI for nuclear counterstaining (Invitrogen). Cell images were acquired with an A1 confocal microscope (Nikon Instrument) using NIS-Elements control software (Version 5.02).

## Supplementary information

Supplementary Information

## Data Availability

Sequence data and counts generated in this study have been deposited to GEO under accession GSE129282.

## References

[1] Settembre C, Fraldi A, Medina DL, Ballabio A (2013). Signals from the lysosome: a control centre for cellular clearance and energy metabolism. Nat Rev Mol Cell Biol.

[2] Dong X-P (2010). PI(3,5)P(2) controls membrane trafficking by direct activation of mucolipin Ca(2+) release channels in the endolysosome. Nature Communications.

[3] Wang X (2012). TPC proteins are phosphoinositide- activated sodium-selective ion channels in endosomes and lysosomes. Cell.

[4] She J (2018). Structural insights into the voltage and phospholipid activation of the mammalian TPC1 channel. Nature.

[5] Kirsch SA, Kugemann A, Carpaneto A, Böckmann RA, Dietrich P (2018). Phosphatidylinositol-3,5-bisphosphate lipid-binding-induced activation of the human two-pore channel 2. Cell. Mol. Life Sci..

[6] Wilson ZN, Scott AL, Dowell RD, Odorizzi G (2018). PI(3,5)P2 controls vacuole potassium transport to support cellular osmoregulation. Mol. Biol. Cell.

[7] Lenk GM, Meisler MH (2014). Mouse models of PI(3,5)P2 deficiency with impaired lysosome function. Meth. Enzymol..

[8] Chow CY (2007). Mutation of FIG4 causes neurodegeneration in the pale tremor mouse and patients with CMT4. J. Nature.

[9] Campeau PM (2013). Yunis-Varón syndrome is caused by mutations in *FIG4*, encoding a phosphoinositide phosphatase. Am. J. Hum. Genet..

[10] Baulac S (2014). Role of the phosphoinositide phosphatase *FIG4* gene in familial epilepsy with polymicrogyria. Neurology.

[11] Lenk GM (2016). Biallelic Mutations of VAC14 in Pediatric-Onset Neurological Disease. Am. J. Hum. Genet..

[12] Shaw JM, Wickner W (1991). T. vac2: a yeast mutant which distinguishes vacuole segregation from Golgi-to-vacuole protein targeting. EMBO J..

[13] Nicolson TA, Weisman LS, Payne GS, Wickner WT (1995). A truncated form of the Pho80 cyclin redirects the Pho85 kinase to disrupt vacuole inheritance in S. cerevisiae. J. Cell Biol..

[14] Gomes de Mesquita DS, van den Hazel HB, Bouwman J, Woldringh CL (1996). Characterization of new vacuolar segregation mutants, isolated by screening for loss of proteinase B self-activation. Eur. J. Cell Biol..

[15] Bonangelino CJ, Catlett NL, Weisman LS (1997). Vac7p, a novel vacuolar protein, is required for normal vacuole inheritance and morphology. Mol. Cell. Biol..

[16] Shalem O, Sanjana NE, Zhang F (2015). High-throughput functional genomics using CRISPR-Cas9. Nat. Rev. Genet..

[17] Ferguson CJ, Lenk GM, Meisler MH (2009). Defective autophagy in neurons and astrocytes from mice deficient in PI(3,5)P2. Hum. Mol. Genet..

[18] Lenk GM (2011). Pathogenic mechanism of the FIG4 mutation responsible for Charcot-Marie-Tooth disease CMT4J. PLoS Genet.

[19] Christensen KA, Myers JT, Swanson JA (2002). pH-dependent regulation of lysosomal calcium in macrophages. J. Cell. Sci..

[20] Ohkuma S, Poole B (1978). Fluorescence probe measurement of the intralysosomal pH in living cells and the perturbation of pH by various agents. Proceedings of the National Academy of Sciences.

[21] Hurwitz SJ, Terashima M, Mizunuma N, Slapak CA (1997). Vesicular anthracycline accumulation in doxorubicin-selected U-937 cells: participation of lysosomes. Blood.

[22] Sanjana NE, Shalem O, Zhang F (2014). Improved vectors and genome-wide libraries for CRISPR screening. Nature Methods.

[23] Schulze U (2014). The Vac14-interaction network is linked to regulators of the endolysosomal and autophagic pathway. Mol. Cell Proteomics.

[24] Jin N (2008). VAC14 nucleates a protein complex essential for the acute interconversion of PI3P and PI(3,5)P(2) in yeast and mouse. EMBO J..

[25] Mole SE, Cotman SL (2015). Genetics of the neuronal ceroid lipofuscinoses (Batten disease). Biochim. Biophys. Acta.

[26] Cárcel-Trullols J, Kovács AD, Pearce DA (2015). Cell biology of the NCL proteins: What they do and don’t do. Biochim. Biophys. Acta.

[27] Chen S (2015). Genome-wide CRISPR screen in a mouse model of tumor growth and metastasis. Cell.

[28] Breslow DK (2018). A CRISPR-based screen for Hedgehog signaling provides insights into ciliary function and ciliopathies. Nature Genetics.

[29] Canver MC (2015). BCL11A enhancer dissection by Cas9-mediated *in situ* saturating mutagenesis. Nature.

[30] Mindell JA (2014). Cell biology. A SWELL channel indeed. Science.

[31] Voss FK (2014). Identification of LRRC8 heteromers as an essential component of the volume-regulated anion channel VRAC. Science.

[32] Syeda R (2016). LRRC8 Proteins Form Volume-Regulated Anion Channels that Sense Ionic Strength. Cell.

[33] Deneka D, Sawicka M, Lam AKM, Paulino C, Dutzler R (2018). Structure of a volume-regulated anion channel of the LRRC8 family. Nature.

[34] Kasuya G (2018). Cryo-EM structures of the human volume-regulated anion channel LRRC8. Nat. Struct. Mol. Biol..

[35] Kefauver JM (2018). Structure of the human volume regulated anion channel. eLife.

[36] Lück JC, Puchkov D, Ullrich F, Jentsch TJ (2018). LRRC8/VRAC anion channels are required for late stages of spermatid development in mice. Journal of Biological Chemistry.

[37] Stuhlmann T, Planells-Cases R, Jentsch TJ (2018). LRRC8/VRAC anion channels enhance β-cell glucose sensing and insulin secretion. Nature Communications.

[38] Ferguson CJ (2012). Neuronal expression of Fig4 is both necessary and sufficient to prevent spongiform neurodegeneration. Hum. Mol. Genet..

[39] He L (2003). Spongiform degeneration in mahoganoid mutant mice. Science.

[40] Lek M (2016). Analysis of protein-coding genetic variation in 60,706 humans. Nature.

[41] Ishida Y, Nayak S, Mindell JA, Grabe M (2013). A model of lysosomal pH regulation. J. Gen. Physiol..

[42] Starita LM (2017). Variant Interpretation: Functional Assays to the Rescue. Am. J. Hum. Genet..

[43] Petersen B-S, Fredrich B, Hoeppner MP, Ellinghaus D, Franke A (2017). Opportunities and challenges of whole-genome and -exome sequencing. BMC Genet..

[44] Sawyer SL (2016). Utility of whole-exome sequencing for those near the end of the diagnostic odyssey: time to address gaps in care. Clin Genet.

[45] Carette JE (2011). Ebola virus entry requires the cholesterol transporter Niemann-Pick C1. Nature.

[46] Kotecki M, Reddy PS, Cochran BH (1999). Isolation and characterization of a near-haploid human cell line. Exp. Cell Res..

[47] Heigwer F, Kerr G, Boutros M (2014). E-CRISP: fast CRISPR target site identification. Nature Methods.

[48] Bae S, Park J, Kim J-S (2014). Cas-OFFinder: a fast and versatile algorithm that searches for potential off-target sites of Cas9 RNA-guided endonucleases. Bioinformatics.

[49] Ran FA (2013). Genome engineering using the CRISPR-Cas9 system. Nature Protocols.

[50] Joung J (2017). Genome-scale activation screen identifies a lncRNA locus regulating a gene neighbourhood. Nature.

