# Supplementary material for: CRISPR knockout screen implicates three genes in lysosome function
Source: Sci Rep. 2019 Jul 3;9:9609. doi: 10.1038/s41598-019-45939-w (PMC6610096; doi:10.1038/s41598-019-45939-w)
Supplement: Supplementary file 1 — Supplementary Information [file 41598_2019_45939_MOESM1_ESM.pdf]

Supplementary Information for

CRISPR knockout screen implicates three genes in lysosome function

Guy M. Lenk[1], Young N. Park[1], Rosemary Lemons[1], Emma Flynn[1], Margaret Plank[1], Christen M. Frei[1], Michael J. Davis[2], Brian Gregorka[2], Joel A. Swanson[2], Miriam H. Meisler[1] and Jacob O. Kitzman[1]

Departments of [1]Human Genetics and [2]Microbiology and Immunology,

University of Michigan, Ann Arbor MI 48109-5618

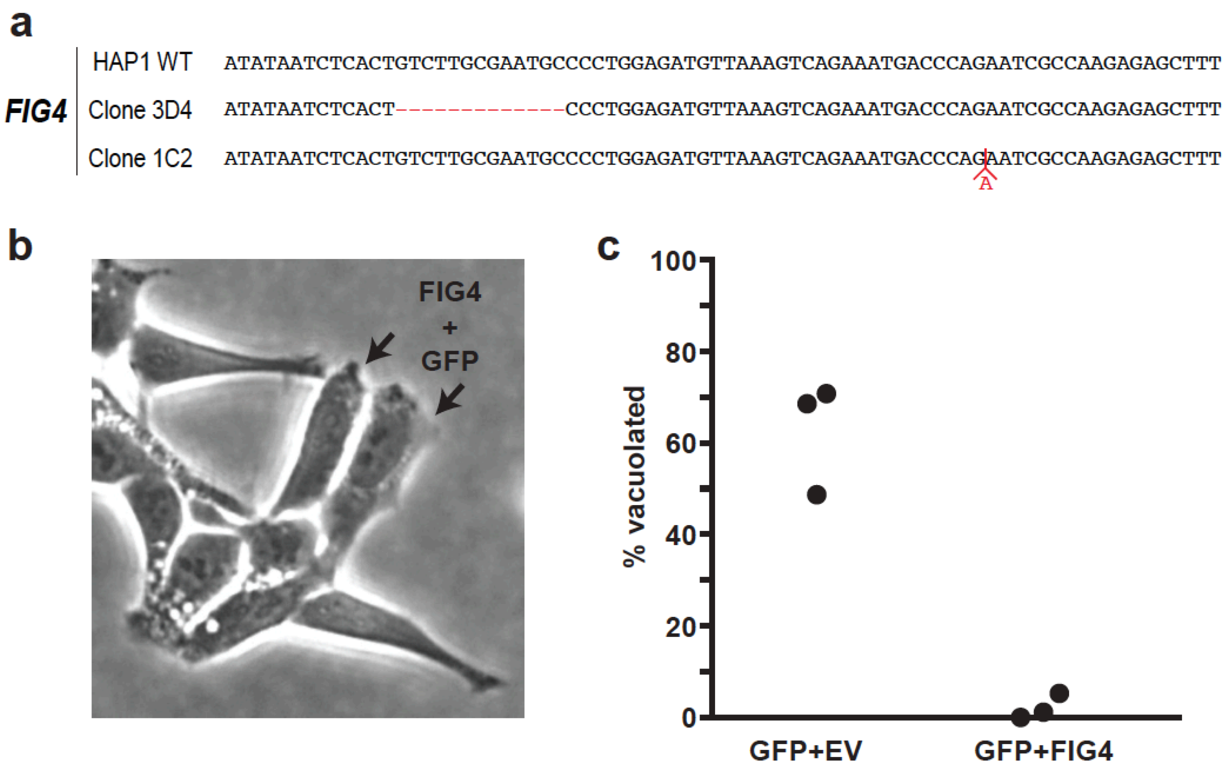

Figure S1: (a) Mutant sequences of FIG4 exon 6 in the FIG4 null clones 1C2 and 3D4. **(b)** Rescue of the enlarged vacuoles in 1C2 cells by transfection of the FIG4 wildtype cDNA. **(c)** The frequency of fluorescent cells which were vacuolated was significantly smaller for the cells co-transfected with FIG4 cDNA (P<2.2x10^-16, Fisher's Exact test).

A

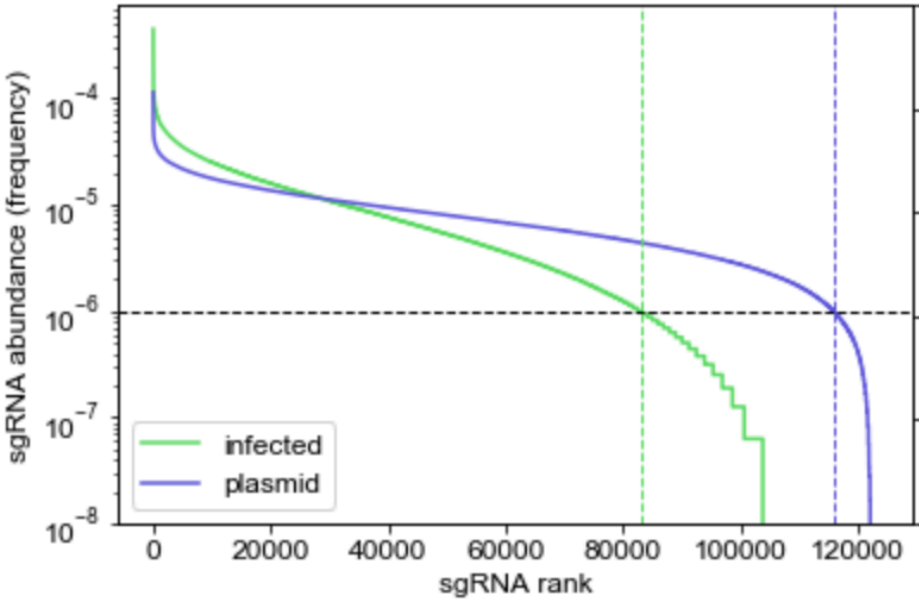

B

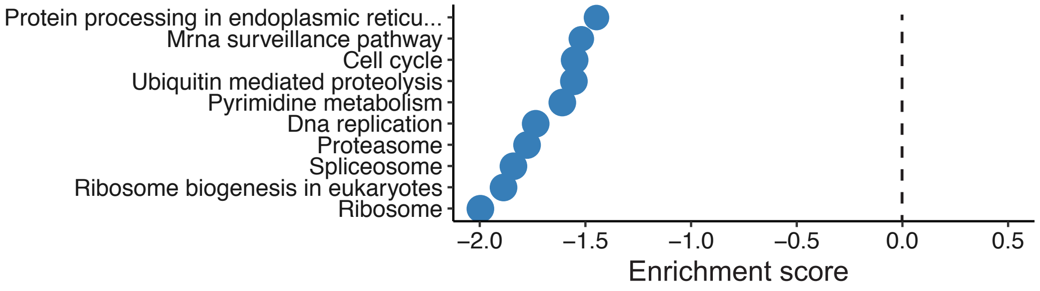

Figure S2. Library screening quality controls. (a). Guide abundances are shown, with frequency of each sgRNA, ranked from most to least abundant, plotted against sgRNA rank for the plasmid pool (blue) and transduced cell population (green). Dotted lines denote numbers of sgRNAs present at frequency of $\geq 10^{-6}$ at each stage. **(b).** Gene categories significantly depleted in surviving transduced cells (fdr-adjusted $p<0.05$) in transduced cells relative to the starting plasmid library (dotted line denotes 0), identified by MAGECK.

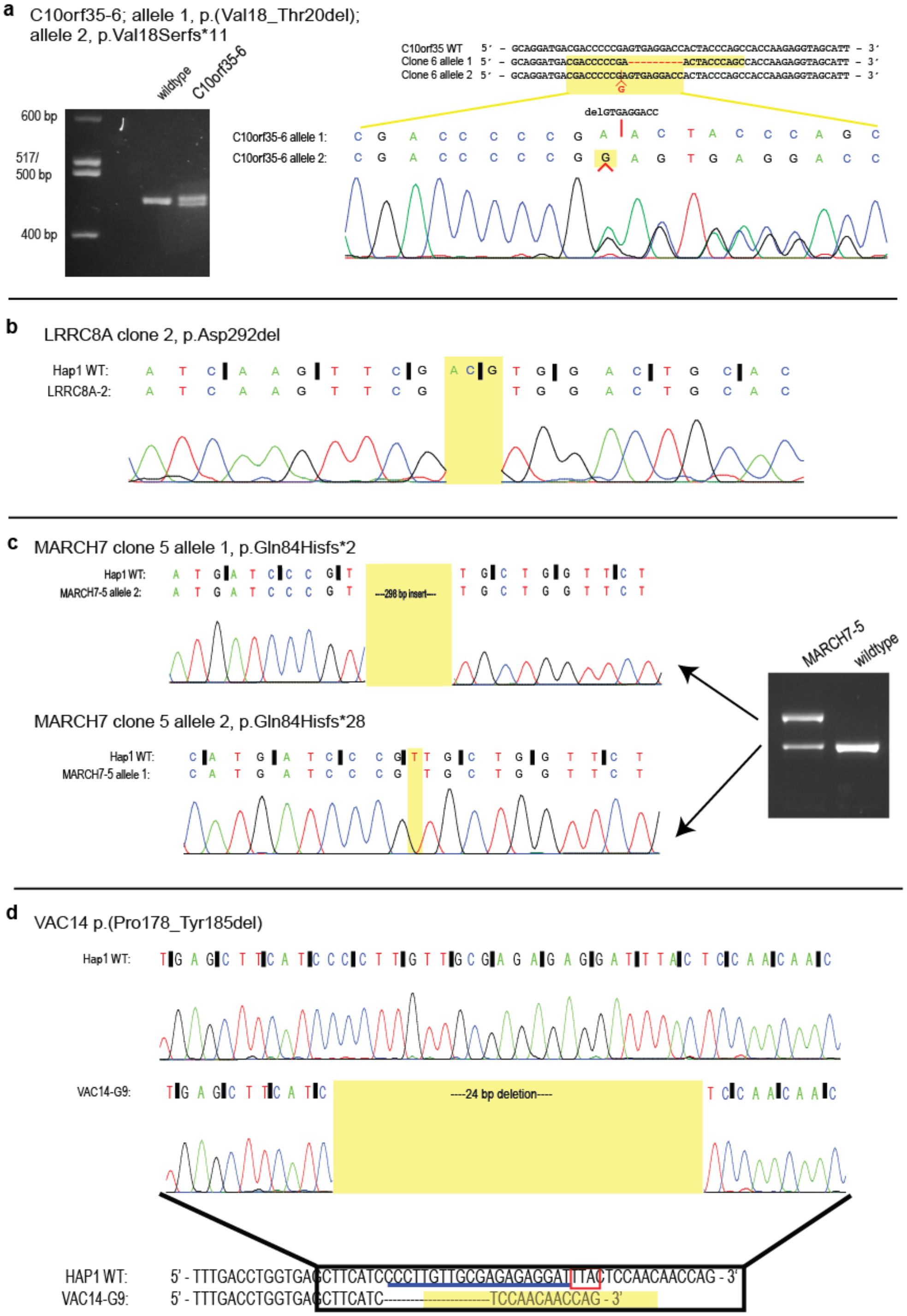

Figure S3. Sequences of the second targeted clones for **(a)** *C10orf35*, **(b)** *LRRC8A*, **(c)** *MARCH7*, and **(d)** *VAC14*. The 9 bp deletion in allele 1 of *C10orf35-6* and the 28 bp insertion in allele 1 of *MARCH7*-5 are visible as doublets after gel electrophoresis. The sequence of the 24 bp deletion in the *VAC14* mutant clone G9 is shown at the bottom of the Figure.

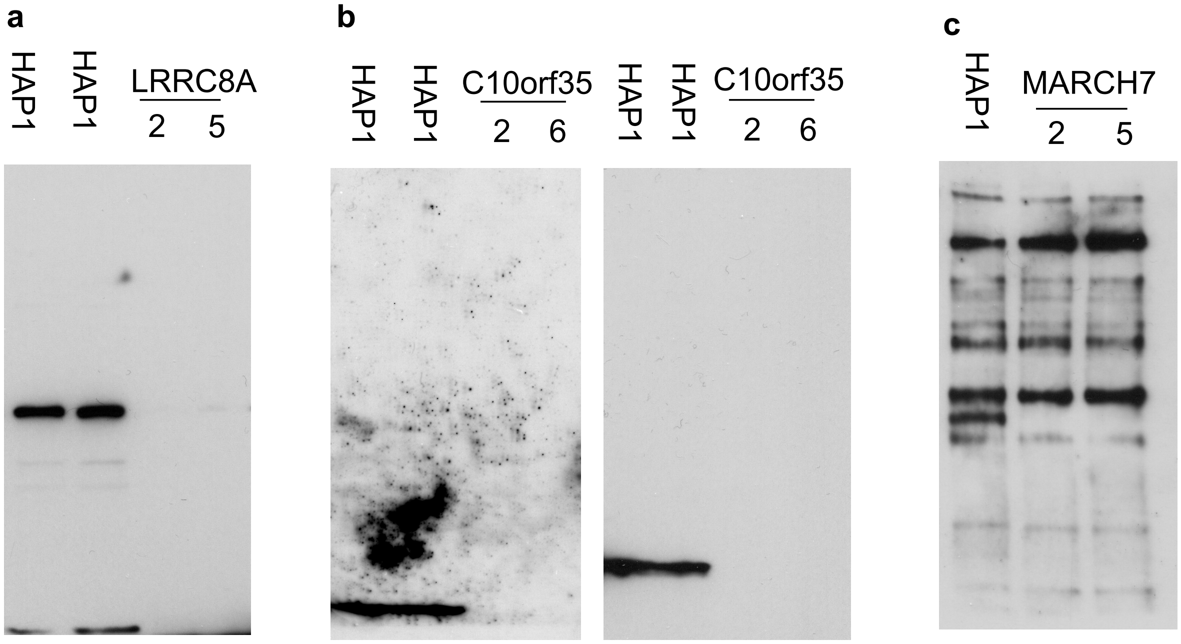

Figure S4. Uncropped Western blot images corresponding to Figure 7 for **(a)** LRRC8A, **(b)** C10orf35, with the original blot on the left and a repeated blot on the right, and **(c)** MARCH7

Table S1. Guide RNAs and PCR primers

Sequence	Use
AAAGCTCTCTTGGCGATTCT	FIG4 guide 1 sequence
CACCAAAGCTCTCTTGGCGATTCT	FIG4 guide 1 top oligo
AAACAGAATCGCCAAGAGAGCTTT	FIG4 guide 1 bottom oligo
CACTGTCTTGCGAATGCCCC	FIG4 guide 2 sequence
CACCCACTGTCTTGCGAATGCCCC	FIG4 guide 2 top oligo
AAACGGGGCATTCGCAAGACAGTG	FIG4 guide 2 bottom oligo
GTGAATCATGATCCCGTTGC	MARCH7 guide 1 sequence
CACCGTGAATCATGATCCCGTTGC	MARCH7 guide 1 top oligo
AAACGCAACGGGATCATGATTCAC	MARCH7 guide 1 bottom oligo
GTTCTGAAGTTCCCGATAAT	MARCH7 guide 2 sequence
CACCGCTTCTGAAGTTCCCGATAAT	MARCH7 guide 2 top oligo
AAACATTATCGGGAACTTCAGAAGC	MARCH7 guide 2 bottom oligo
GGGTAGTGGTCCTCACTCGG	C10orf35 guide 1 sequence
CACCGGGTAGTGGTCCTCACTCGG	C10orf35 guide 1 top oligo
AAACCCGAGTGAGGACCACTACCC	C10orf35 guide 1 bottom oligo
GATCCTGCACGATTTCCCCAT	C10orf35 guide 2 sequence
CACCGATCCTGCACGATTTCCCCAT	C10orf35 guide 2 top oligo
AAACATGGGGAAATCGTGCAGGATC	C10orf35 guide 2 bottom oligo
GCACAACATCAAGTTCGACG	LRRC8A guide 1 sequence
CACCGCACAACATCAAGTTCGACG	LRRC8A guide 1 top oligo
AAACCGTCGAACTTGATGTTGTGC	LRRC8A guide 1 bottom oligo
GCCTGACGCAGATCGAGCTG	LRRC8A guide 2 sequence
CACCGACCTGACGCAGATCGAGCTG	LRRC8A guide 2 top oligo
AAACCAGCTCGATCTGCGTCAGGTC	LRRC8A guide 2 bottom oligo
GAATCCAGCAGGTTAATGTC	VAC14 guide 1 sequence
CACCGTAATCCAGCAGGTTAATGTC	VAC14 guide 1 top oligo
AAACGACATTAACCTGCTGGATTAC	VAC14 guide 1 bottom oligo
GCATCCCCTTGTTGCGAGAG	VAC14 guide 2 sequence
CACCGTCATCCCCTTGTTGCGAGAG	VAC14 guide 2 top oligo
AAACCTCTCGCAACAAGGGGATGAC	VAC14 guide 2 bottom oligo
5'-CTAAATGGCTGTGAGAGAGCTCAG-[N_{9-11}]-TCTTGTGGAAAGGACGAAACACCG-3'	Modified GECKO forward PCR primer
